# Microbial Response to Experimentally Controlled Redox Transitions at the Sediment Water Interface

**DOI:** 10.1371/journal.pone.0143428

**Published:** 2015-11-24

**Authors:** Katharina Frindte, Martin Allgaier, Hans-Peter Grossart, Werner Eckert

**Affiliations:** 1 Department of Soil Science, Institute for Crop Science and Resource Conservation (INRES), University of Bonn, Nußallee 13, Bonn, Germany; 2 Berlin Center for Genomics in Biodiversity Research (BeGenDiv), Königin-Luise-Straße 6-8, Berlin, Germany; 3 Leibniz-Institute of Freshwater Ecology and Inland Fisheries, Alte Fischerhütte 2, Stechlin, Germany; 4 Institute of Biochemistry and Biology, University of Potsdam, Am Neuen Palais 10, Potsdam, Germany; 5 Israel Oceanographic and Limnological Research, The Yigal Allon Kinneret Limnological Laboratory, Migdal, Israel; Auckland University of Technology, NEW ZEALAND

## Abstract

The sediment–water interface of freshwater lakes is characterized by sharp chemical gradients, shaped by the interplay between physical, chemical and microbial processes. As dissolved oxygen is depleted in the uppermost sediment, the availability of alternative electron acceptors, e.g. nitrate and sulfate, becomes the limiting factor. We performed a time series experiment in a mesocosm to simulate the transition from aerobic to anaerobic conditions at the sediment–water interface. Our goal was to identify changes in the microbial activity due to redox transitions induced by successive depletion of available electron acceptors. Monitoring critical hydrochemical parameters in the overlying water in conjunction with a new sampling strategy for sediment bacteria enabled us to correlate redox changes in the water to shifts in the active microbial community and the expression of functional genes representing specific redox-dependent microbial processes. Our results show that during several transitions from oxic-heterotrophic condition to sulfate-reducing condition, nitrate-availability and the on-set of sulfate reduction strongly affected the corresponding functional gene expression. There was evidence of anaerobic methane oxidation with NO_x_. DGGE analysis revealed redox-related changes in microbial activity and expression of functional genes involved in sulfate and nitrite reduction, whereas methanogenesis and methanotrophy showed only minor changes during redox transitions. The combination of high-frequency chemical measurements and molecular methods provide new insights into the temporal dynamics of the interplay between microbial activity and specific redox transitions at the sediment–water interface.

## Introduction

The sediment–water interface (SWI) of aquatic systems is characterized by biogeochemical gradients and exchange processes between liquid and solid phases. Diffusion gradients in conjunction with near-bottom currents restrict mixing between porewater and the overlying water, resulting in sharp redox gradients within a few millimeters [1]. The environmental conditions at this interface can be highly dynamic in space and time due to complex interactions between abiotic and biotic factors. For example, oxygen depletion in the uppermost sediment layer results in changes in heterotrophic and chemolithotrophic microbial activities, as well as chemical oxidation of upward diffusing reduced solutes such as methane and sulfide [2,3]. Below the oxycline, facultative anaerobic microorganisms use nitrate, nitrite, or ferric iron as electron acceptors. Further down, strictly anaerobic microorganisms, which are capable of using less favorable electron acceptors such as sulfate and carbon dioxide, prevail [4]. At the SWI of natural lakes, this transition occurs on a time scale of minutes to months. Short-term fluctuations in the oxycline occur as a consequence of changing near-bottom currents affecting diffusion processes in the sediment porewater [5,6]. Temporary, but locally restricted, oxygenation of anoxic sediments can be induced by bioturbation (e.g., animal burrows), or on a day–night basis by macrophyte roots and benthic primary production [7–9]. In thermally stratified lakes with an anoxic hypolimnion, internal wave activity can cause periodic exposure of bottom sediments to alternating oxic–anoxic conditions as a function of wave amplitude and frequency [10,11]. Little is known regarding the immediate impact of these redox changes on microbial activities at the SWI and vice versa.

Previous studies have shown that the total sedimentary microbial community structure changed only mildly in relation to redox gradients [12,13]. Similarly, Tšertova et al. [14,15] concluded that community composition of sediment microorganisms was less affected by changes in the availability of electron acceptors.

The goal of the present study was to expand the analysis of redox-related microbial responses from the community level to instantaneous activity changes of sediment bacteria. In a mesocosm study with an intact sediment core from the oligotrophic Lake Stechlin (NE Germany), we exposed the SWI to a series of redox changes while following in parallel the expression of specific functional genes in conjunction with the composition of the active microbial community at the SWI as revealed by 16S rRNA analysis. We hypothesize that redox shifts will more strongly affect functional gene expression than community composition of active sediment microorganisms. We also introduce a novel resuspension-sampling technique for subsampling both water- and sediment-associated microorganisms.

## Methods

### Study Site

The oligotrophic Lake Stechlin is located in the Brandenburg-Mecklenburg Lake District, Germany (53°10’ N, 13°02’ E). Field permit was granted to the Leibniz-Institute of Freshwater Ecology and Inland Fisheries by the Stechlin Park nature authorities on a permanent basis. Lake Stechlin is a hard water lake which encloses an area of 4.25 km^2^ with a maximum and mean depth of 68 m and 22.8 m, respectively. Average total phosphorus concentration is on average 13.1 μg L^-1^ during summer time [16]. The entire water column remains oxic throughout the year, but in the sediments dissolved oxygen is depleted in the uppermost layers. The lake has a relatively high sulfate concentration of 200 μmol L^-1^ in the water column. In the sediment, the upper 7 cm is depleted in sulfate and is characterized by elevated porewater sulfide concentrations [17]. Dumont et al. [18] found relatively low methane uptake rates under *in situ* conditions. Methane production primarily occurs in deeper sediment layers (20–25 cm), but could potentially take place in surface layers as well [19]. Denitrification is relatively low in the littoral sediments, but higher at the lake’s center [20]. We used profundal sediments for the experiments to obtain maximum microbial activity. The sediments at the lake’s centre were most promising in this regard due to their fine texture and relatively high organic matter content.

### Experimental design

**Fig 1:**
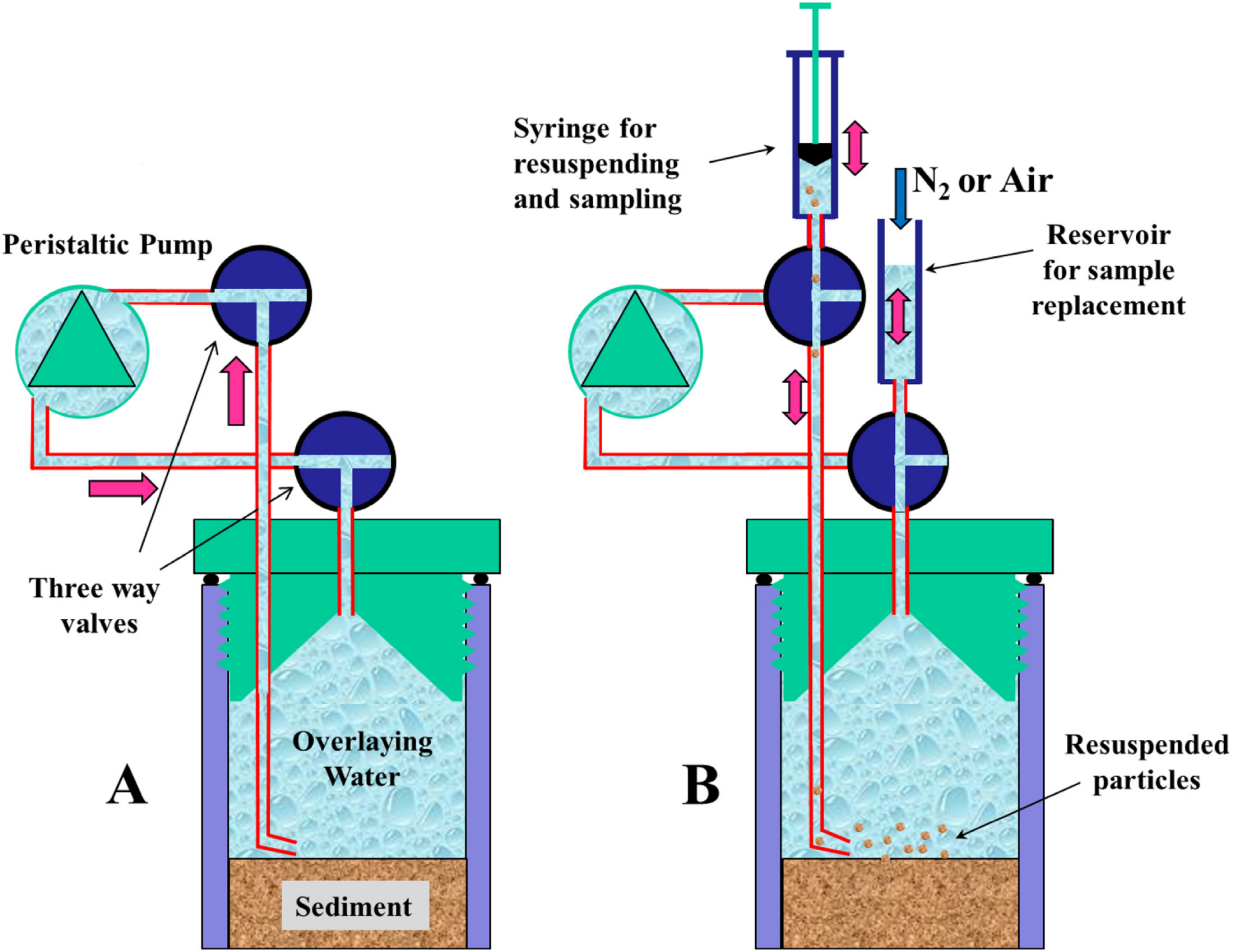
Experimental design without electrodes. (A) Valve settings during circulation via pumping to prevent a formation of diffusive boundary layer above the sediment and to ensure an equal distribution of solutes in the water column; (B) Valve settings during resuspension /sampling mode with pump off.

In June 2010, a 40 cm long, intact sediment core with an overlying water column of 20 cm was taken from the deepest point of the lake with a gravity corer (UWITEC, Mondsee, Austria). The core was hermetically sealed and mounted as shown in Fig 1A and 1B (without the electrochemical sensors). The overlying water was constantly circulated by a peristaltic pump (Abimed) to prevent chemical stratification. The core was sealed with airtight caps to exclude external oxygen. Incubation experiments were performed at room temperature (22–25°C) rather than the *in situ* temperature (6–7°C) so that changes in microbial activities would be more readily observable within the experimental period. Likewise, we added a high initial concentration of potassium nitrate (~3 mmol L^-1^) to the oxic overlaying water to increase the redox buffer capacity. Goal of the prolonged presence of nitrate was to delay the subsequent processes in a way that rendered optimum timing of our subsampling. In the course of the experiment we repeatedly added oxygen (air bubbling) and/or potassium nitrate to check for microbial responses after the depletion of the particular electron acceptor.

### Chemical measurements

In order to follow the hydrochemical changes in the overlaying water in real time, dissolved oxygen (DO), pH, oxidation–reduction potential (ORP) and hydrogen sulfide were monitored online as previously described [21]. Nitrate was measured by an ion-selective nitrate electrode (Mettler-Toledo, Gießen, Germany). Dissolved methane in the overlying water was measured daily using a modified version of the head space technique [22]. Two 1 ml subsamples were transferred into 10 ml crimp vials prefilled with N_2_. After sealing the vials, dissolved CH_4_ was extracted into the gas phase by vigorous shaking and, thereafter, analysed on a gas chromatograph with a flame ionization detector (Shimadzu, Duisburg, Germany).

### Microbiological analysis

#### Sampling

A technique was developed that allowed us to repeatedly sample the overlying water together with the sediment-associated microbes with minimal disturbances. In this approach (Fig 1), the uppermost sediment layer was resuspended by gently agitating the SWI with pulses of turbulence induced via a syringe connected to a tube (diameter: 3 mm) with an angular outlet positioned a few millimeters above the sediment. A second syringe filled with pre-conditioned (oxic or anoxic) water ensured the quantitative replacement of the sampled water volume. The use of two three-way-valves within the pump cycle enabled switching between circulation mode and sampling mode, a design that proved to be ideal for preventing oxygen contamination. Samples containing resuspended sediment particles and overlaying water (ca. 10 mL) were filtered directly onto 0.2 μm polycarbonate filters and transferred immediately into liquid nitrogen. This set-up further allowed collection of a high number of subsamples during the incubation experiment. This would not be possible with conventional experimental designs including rhizotrones, which only collect bacteria in the sediment porewater and also affect the sediment depth. All microbiological samples were immediately stored at -80°C until further processing. Sampling time points were chosen in relation to the imposed redox changes, and varied between twice per day to once every second day.

### RNA extraction and cDNA synthesis

RNA for studying community composition of active microorganisms was extracted from samples collected in the experiment. We used chloroform-phenol-isoamyl alcohol and zirconium beads following the protocol of Nercessian et al. [23], except that the extraction buffer was prepared with 240 mM instead of 200 mM potassium phosphate buffer. The DNA- RNA-pellet was dissolved in 35 μL RNase-free water and stored at -80°C. DNA was digested twice with Turbo-DNA free kit (Ambion) following the manufacturer’s instructions, except that 2.5 μL instead of 2 μL of the reaction buffer was used. Successful removal of DNA was checked by PCR using the universal primer pair 341f/ 907r (S1 Table). Approximately 100 ng of total RNA was used for cDNA synthesis via the Array Script kit (Ambion). However, we were unable to produce cDNA from some of the samples, especially for sample points between 250–400 h and 500–600 h.

### Polymerase chain reactions

The reaction mixtures for PCR amplifications contained 5–20 ng μL^-1^ cDNA, 0.4–0.8 pmol μL^-1^ of each primer (S1 Table), 250 μmol L^-1^ of each dNTP, 3 mmol L^-1^ MgCl, 2.5 μL of 10 × PCR buffer, and 0.5 U Biotaq Red DNA polymerase (Bioline) in a total volume of 50 μL. In addition to the universal *Bacteria* and *Archaea* primers, we used a set of primers suitable for amplifying genes of some key enzymes in elemental cycling. Unfortunately, the partial sequence of the ammonium monooxygenase gene (amoA) from bacteria and archaea could not be successfully amplified in a PCR approach. All successfully amplified genes and their functions are given in Table 1. Primer sequences and PCR conditions are provided in S1 Table.

**Table 1:** Applied PCR approaches and the function of their target genes.

16S and functional genes	Function	Related processes
Bacteria 16S rRNA	Coding for bacterial small ribosomal subunit RNA	
Archaea 16S rRNA	Coding for archaeal small ribosomal subunit RNA	
Ammonium Oxidizers 16S rRNA	Coding for bacterial small ribosomal subunit RNA of ammonium oxidizers	
dsrB	Dissimilatory sulfite reductase—Dissimilatory sulfate reduction	Sulfate reduction
aprA	Dissimilatory adenosine-5-phosphosulfate reductase Dissimilatory sulfate reduction and sulfide oxidation	Sulfate reduction—Sulfide oxidation
mcrA	Methyl-coenzyme M reductase—Methanogenesis	Methanogenesis
nirS	Cytochrome cd_1_-depending nitrite reductase Dissimilatory nitrite reduction	Denitrification
nirK	Copper-depending nitrite reductase—Dissimilatory nitrite reduction	Denitrification
pmoA	Particulate methane monooxygenase—Methane oxidation	Methane oxidation

#### Denaturing gradient gel electrophoresis

Denaturing gradient gel electrophoresis (DGGE) was used to determine diversity and dynamics of the active microbial communities present in the experimental sediment core [16]. All DGGEs were performed with the PhorU system (Ingeny, Goes, Netherlands), but with different denaturing gradients (S1 Table). All gels were stained with SybrGold.

#### Sequencing of DGGE-bands

Most prominent DGGE bands and bands reflecting shifts in the microbial community were excised and resolved in 20 μL TE buffer for sequencing. The bands were re-amplified using the appropriate primers as described in S1 Table. PCR products were purified by 1:1 addition of a precipitation solution (20% polyethylenglycol 8000 and 2.5 mol L^-1^ NaCl in distilled water). PCR products were incubated for 20 min, and thereafter centrifuged (at 17000 g). The pellet was washed with 100 μL 70% ethanol. Sequencing was performed on an ABI Sequencer 3130 (Applied Biosystems, Darmstadt, Germany) following the manufacturer’s instructions.

### Data analysis

#### Cluster analysis and statistics

Cluster analysis of DGGE banding patterns was performed in GelCompar II (Applied maths, Sint-Martens-Latem, Beligium) using Dice similarity measures. Spearman’s rank correlation between methane and nitrate, non-metric multidimensional scaling (NMDS) and envfit analyses were performed in R (2009–2011 RStudio Inc., Version 3) to evaluate six environmental parameters (sulfide, methane, pH, nitrate, DO and redox potential) in relation to the microbial community as determined by band diversity in DGGE gels. All results were corrected using Bonferroni-Holm correction.

#### Phylogenetic analysis

16S rRNA sequences were checked for chimera with DECIPHER; chimeras and uncertain sequences were then removed [24]. Phylogenetic analysis of the 16S rRNA gene fragments and functional genes was performed using the ARB software package (http://www.arb-home.de; [25]). The 16S rRNA sequences (sequence length: ca. 450 bp for *Bacteria* or ammonium oxidizers and 300 bp for *Archaea*) were pre-aligned using the SINA aligner (http://www.arb-silva.de/aligner) and imported into the SILVA database (version SILVA 108 SSU 111 database) for further analysis. Potential alignment errors were corrected manually. For stability of the phylogenetic tree a backbone tree was calculated with sequences of at least 1,200 nucleotides using default settings of different maximum likelihood approaches provided within ARB (RAxML and PHYML). Sequence fragments from our study were added to this tree afterwards according to maximum parsimony criteria. This method does not correct for evolutionary distances and does not allow for changes in the overall tree topology. We used the base frequency filter for bacteria provided in the SILVA database to exclude highly variable positions for our phylogenetic reconstructions. The final tree was calculated using RAxML. All sequences can be found in Genbank (KT326705-326765).

Sequences of the different functional genes were quality checked and translated into amino acid sequences for phylogenetic analysis. Phylogenetic relatives were found by using the Blast tool (http://blast.ncbi.nlm.nih.gov). All sequences are submitted to GenBank (KT327082-327131 and KT933256-KT933330).

## Results

### Transition phase 1: From oxygen to nitrate (0–130 h)

**Fig 2:**
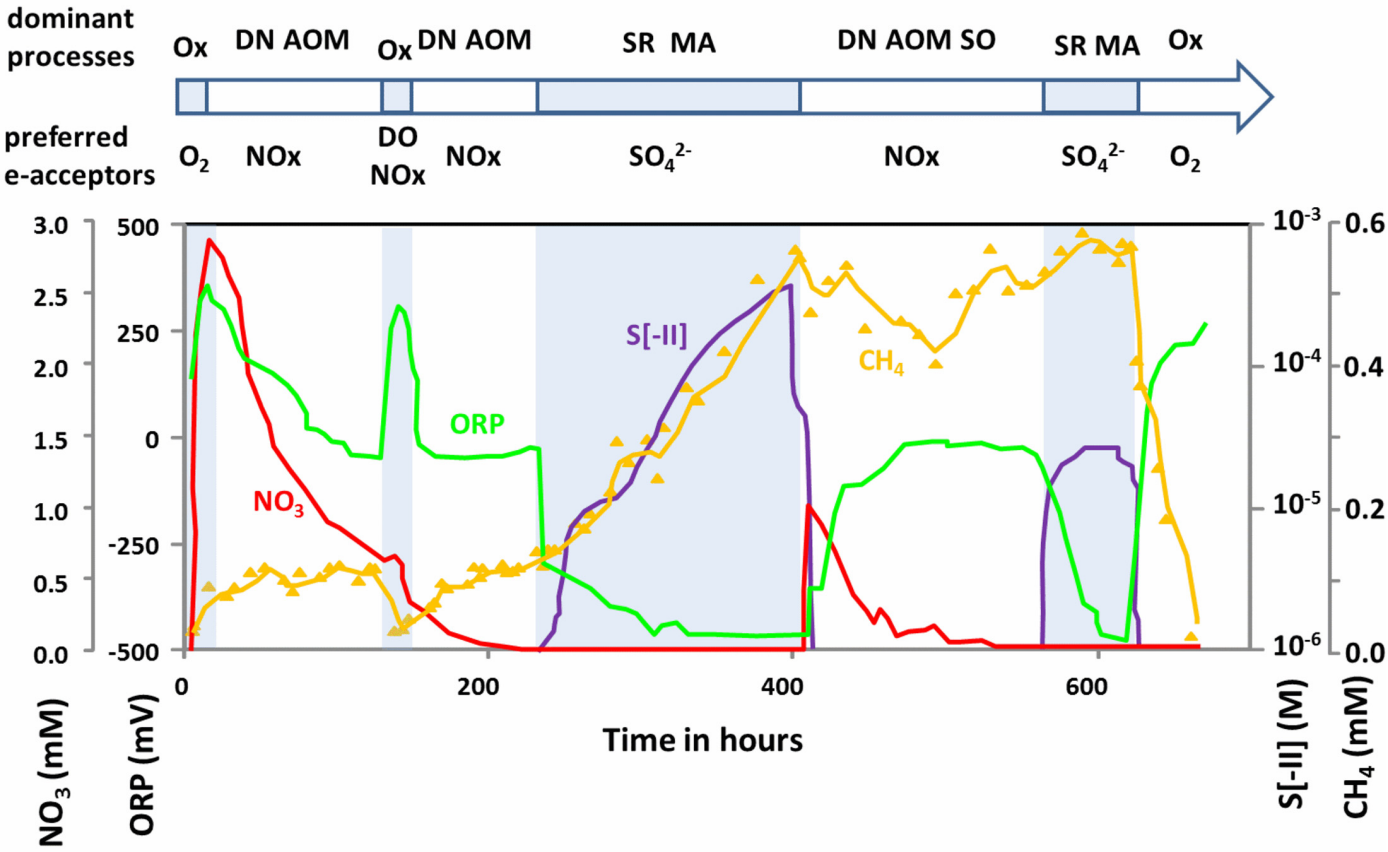
Hydrochemical changes in the overlying water of an incubated sediment core during redox succession. The time line above indicates the different microbial phases within the experiment and highlights the preferred electron acceptors within these phases. Ox: Oxygen consumption; DN: Denitrification; AOM: anaerobic oxidation of methane; MA: Methane Accumulation; SR: Sulfate Reduction; SO: Sulfide oxidation.

**Fig 3:**
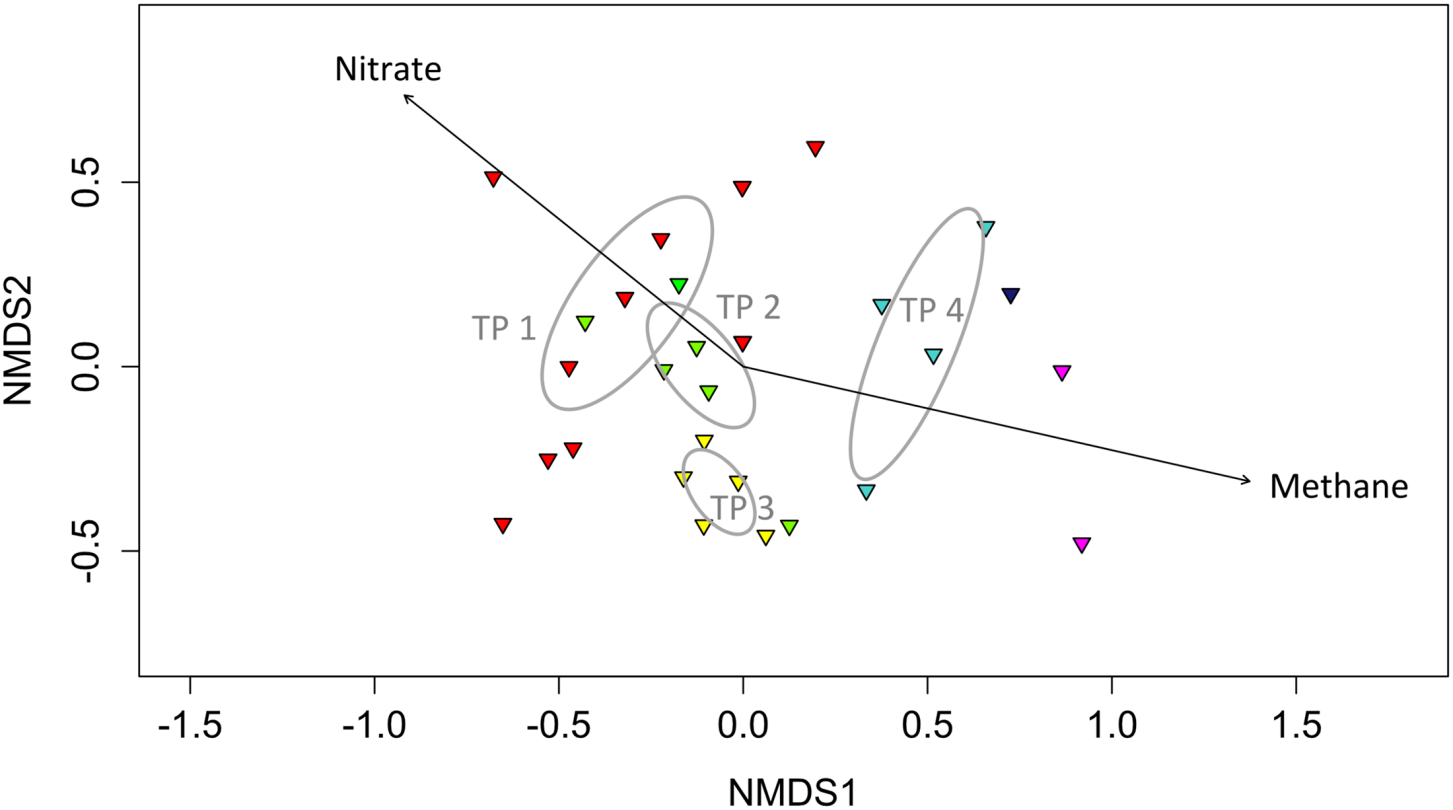
NMDS plot (stress 0.13) of 16S rRNA bacterial DGGE banding patterns. Arrows indicate the direction triggered by methane and nitrate as the strongest analyzed environmental factors. Ellipses indicate a confidence interval of 95% and ANOSIM analysis was significant (p = 0.001, r = 0.558). Colors indicate the six transitions phases (TP): red = TP 1 (Transition from oxygen to nitrate as electron acceptor), green = TP 2 (Short oxic pulse), yellow = TP 3 (nitrate depletion and sulfide development), light blue = TP 4 (Nitrate additions under sulfidic condition), dark blue = sulfidic phase, purple = oxic phase.

**Fig 4:**
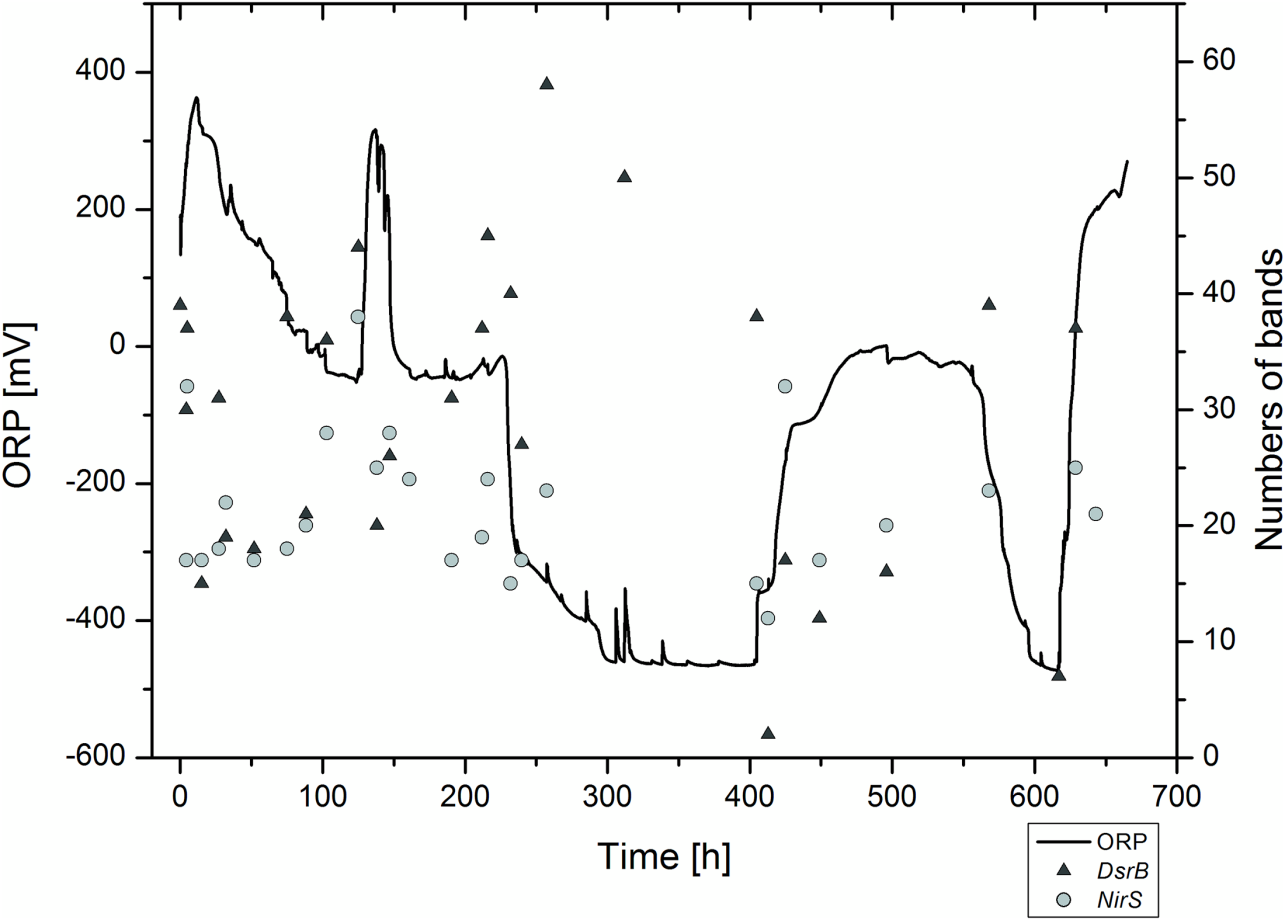
Development of *dsrB* and *nirS* DGGE band diversity in relation to ORP.

The initial overlying water was oxic with an ORP of 150 mV (Fig 2). After four hours of incubation we added the nitrate redox buffer, which raised the ORP to 380 mV. The initial 150 μM of dissolved oxygen (DO, not shown in Fig 2) in the overlying water was depleted within the first 10 h after the system was sealed off from external air. At the SWI, the depletion of DO triggered the first transition phase from oxygen as the electron acceptor to nitrate. This phase lasted for about 130 h and was accompanied by a gradual ORP decrease to -40 mV. DGGE 16S rRNA fingerprint analysis displayed a shift in the microbial community composition after 50 h: two prominent bands disappeared during the transition from oxic to anoxic conditions (S1 Fig). NMDS analysis showed a high variance within the first transition phase and no clear selection from the second transition phase (Fig 3). ANOSIM analysis showed significant differences between the clusters (p = 0.001, r = 0.558). Envfit analysis suggests methane and nitrate, but not redox potential, as significant triggers (p = 0.002 and 0.007, see S2 Table). The main environmental influences on DGGE patterns of “active” microorganisms and expressed functional genes must be interpreted carefully due to covariance of various chemical parameters in the sediments. DGGE analysis of active *Archaea* (16S rRNA), however, did not show any prominent change (S2 Fig). Expression patterns of the *nirS* gene for denitrification significantly changed after oxygen depletion and after 32 h when nitrate concentrations dropped below 2.0 mmol L^-1^ and ORP was below 200 mV (S3A Fig). This was consistent with a preliminary experiment demonstrating rapid shifts in *nirS* expression in response to nitrate addition within one hour (Cluster analysis; S4 Fig). The expression pattern of *nirS* displayed a high diversity while band diversity of *nirK* was much lower (S3B and S5 Figs). Sulfate reducers and methanogenic archaea were detectable under oxic and denitrifying conditions. Although the sulfate reducing community (represented by *dsrB* expression patterns) revealed significant shifts between time points, no clear trend was observed during the transition from oxygen to nitrate as electron acceptor (Fig 4, S3A Fig). After the depletion of DO, dissolved methane increased from 0.02 to 0.1 mmol L^-1^ within 48 hours, and remained at this level until reoxidation of the overlying water (hour 130). Active methanogenic archaea could be detected with 16S rRNA as well as *mcrA* specific primers at all times. DGGE pattern of archaeal 16S rRNA changed significantly due to ORP (p = 0.001, see S1 Table) but only in non-dominant bands (S2 Fig). *McrA* DGGE pattern was not influenced by the measured environmental parameters (oxygen: p = 0.04, but did not remain significant after Bonferroni-Holm correction, S2 Table). This indicates that ORP did not play a crucial role in shaping the community of active *Archaea* and particularly methanogens at the SWI. In contrast, *pmoA* expression (representing active methanotrophy) was detected repeatedly during the experiment without using a nested PCR approach as long as oxygen and/or nitrate was present. This suggests that aerobic oxidation of upward diffusing methane prevented methane accumulation in the overlying water. As long as nitrate was present in the overlying water, anaerobic oxidation of methane with nitrate (AOM) limited CH_4_ increase to 0.1 mmol L^-1^. Spearman's rank correlation analysis showed that nitrate and methane values are negatively related (r = -0.53, p < 0.001). Similar trends in chemical parameters were observed in a preliminary experiment in 2007 (S6 Fig). Sequence analysis of active methanotrophs revealed no changes in community composition, despite the absence of oxygen.

### Transition phase 2: Reaction to DO pulse (130–230 h)

**Fig 5:**
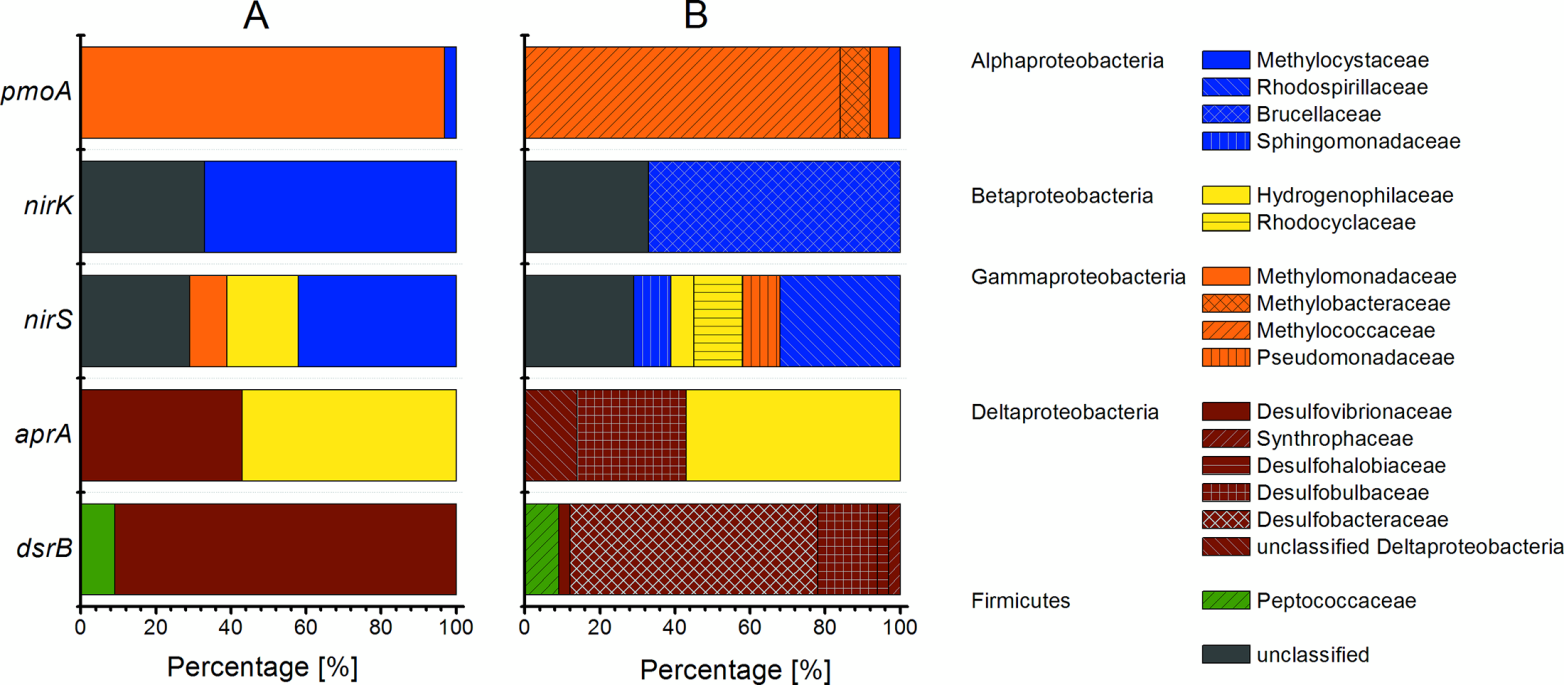
Bar chart of analyzed functional genes and their phylogenetic classification. (A) Phylum-level classification of functional gene sequences; (B) Phylogenetic classification of the sequences to their closest related family. Colors correspond to encountered phyla, while patterns indicate families.

After 130 h of incubation, we added 165 μmol L^-1^ oxygen to the overlying water. As a result ORP increased rapidly to 300 mV and dissolved methane dropped to 0.02 mmol L^-1^. The occurrence of oxic processes was not reflected by changes in DGGE banding patterns of the active bacterial community. NMDS analysis, however, revealed a second cluster corresponding to the second transition phase including the samples taken when nitrate was below the detection limit (130–230 h; Fig 3). *Magnetospirillum* appeared to be the predominant species with up to three distinct bands (185–240 h; S1 Fig). We found no clear reaction of the active microbial community to the oxygen pulse, which lasted only for about 15 h when ORP dropped from ca. 300 mV back to its previous level of -40 mV. Methane increased again from 0.01 to 0.1 mmol L^-1^. Two previously dominant 16S rRNA bands from the first oxic phase vanished after oxygen depletion and did not reoccur despite subsequent oxygen additions (S1 Fig, red squares). Nitrate remained detectable only up to 200 h, but ORP suggests that it was still present below detection (<0.01 mmol L^-1^) until 230 h. The *nirS* expression pattern (denitrification) changed after 146 h in the absence of nitrate, indicating changes within the active denitrifying community. Expression patterns of *mcrA* (methanogenesis) and *nirK* (denitrification) genes in relation to the environmental parameters did not change significantly throughout the whole experiment (see S2 Table). Interestingly, there seems to be a slight effect on *mcrA* band diversity, since band number was highest at transition phases, which was not the case for *nirK* (S5 Fig). *AprA* (sulfide oxidation and sulfate reduction) and *pmoA* (methanotrophy) genes exhibited changes which could be related to fluctuations in ORP, (p = 0.011), methane (p = 0.001) and pH (p = 0.006). For *pmoA*, 31 of the 37 sequences were affiliated with methanotrophic type Ib *Methylococcaceae* occurring at different heights on the DGGE gel. Only four of the identified *pmoA* sequences in this study belonged to the type Ia methanotrophs (Fig 5). Only one sequence was classified as methanotrophic bacteria type II (*Methylocystis)* which was detected only under oxic conditions.

### Transition phase 3: Depletion of nitrate and first sulfidic phase (230–400 h)

With the depletion of nitrate at 230 h, the ORP signal dropped rapidly from 0 to -250 mV and then gradually to -480 mV (Fig 2). From that time point onwards sulfide concentration increased in the overlying water. Interestingly, dissolved methane that was absent under oxic condition and present at low levels before the depletion of nitrate, increased in parallel to sulfide concentration. With the ORP dropping below -200 mV, bacterial community composition showed a third cluster in the NMDS (Fig 3). During this phase, *Magnetospirillum* appeared to be the predominant bacterial phylotype occurring in the 16S rRNA DGGE gel (S2 Fig). Sulfate reducers (*dsrB* expression patterns) showed a strong increase in diversity when the ORP dropped from -40 to -500 mV, which was not the case for *nirS* (Fig 4). The main DGGE bands in *nirK* expression patterns vanished between 210–230 h, and three additional bands occurred exclusively during the following transition phase. After the transition phase, *nirK* expression patterns were similar to those before the transition, although they seemed to be less diverse. At 400 h both methane and total sulfide reached concentrations of 0.5–0.6 mmol L^-1^.

### Transition phase 4: Nitrate addition under sulfidic conditions (400–570 h)

Nitrate was added a second time at 400 h to a final concentration of 1 mmol L^-1^. As a consequence, sulfide was oxidized completely within 30 h, and methane concentrations declined from 0.55 mmol L^-1^ at 400 h to 0.4 mmol L^-1^ at 500 h when nitrate concentration dropped below the detection limit (Fig 2). Interestingly, ORP increased in the presence of nitrate to the same values (~ -40 mV) as during the previous nitrate addition in the absence of DO. The observed hydrochemical changes led to a physiological response as indicated by the fourth, distinctly different cluster in the NMDS analysis based on 16S rRNA DGGE banding patterns (Fig 3). The *dsrB* expression pattern changed with nitrate, as indicated by a sudden decrease in DGGE band diversity (Fig 4, S3 Fig). Denitrifiers expressing *nirS* did not show major changes in DGGE banding patterns upon the second nitrate addition. DGGE bands that were prominent after the first nitrate addition did not reoccur. In line with the decrease in methane, *pmoA* could be amplified again without using a nested PCR approach.

Interestingly, *aprA* genes affiliated to *Hydrogenophilaceae* were expressed when sulfide was present, but were not detectable when nitrate concentrations were high and sulfide not detectable. After nitrate depletion (570 h), *aprA* genes of the organism were expressed again. In contrast, the prominently expressed *aprA* genes associated with *Deltaproteobacteria* were always detectable between 230–640 h, i.e. the first depletion of nitrate until the reintroduction of DO.

### Transition phase 5: Second sulfidic phase (570–630 h)

Right after the depletion of nitrate at ~570 h, sulfide and methane concentrations increased to 0.03 and 0.58 mmo L^-1^, respectively, while ORP dropped back to -500 mV (Fig 2). Analysis of a single sample taken during the prominent ORP drop with increasing sulfide concentrations confirmed our results from the first sulfate reduction phase. At that time, expressed *dsrB* gene diversity strongly increased during the transition from oxidized nitrogen species to sulfate as the electron acceptor (Fig 4, S3A Fig). In contrast, expressed *aprA* gene pattern did not change during this transition phase.

### Transition phase 6: third oxic phase (> 630 h)

At T = 630 h, the overlying water was turned oxic again, leading to the rapid oxidation of methane and sulfide. Apparently this drastic redox change did not result in measureable effects on the expression of functional genes or the composition of active microorganisms (Fig 2, S1 Fig). These results match observations made in the aftermath of the DO addition at 130 h.

### Composition of the active microbial community

Most of the active bacteria community belonged to *Betaproteobacteria* (56%), while only a few sequences belonged to *Alphaproteobacteria* (16%), *Bacteriodetes* (6%), *Cyanobacteria* (9%) and *Epsilonproteobacteria* (3%) (S7A Fig). Several DGGE bands of bacteria that were present over the course of the experiment were identified as members of *Caulobacteraceae* and *Rhodospirillaceae*.

16S rRNA DGGE analysis did not reveal any *Delta*- or *Gammaproteobacteria* affiliated with *aprA*, *dsrB* and *pmoA*. Phylogenetic classification of 7 *aprA* and 32 *dsrB* sequences indicate that these sequences were affiliated mainly with *Deltaproteobacteria* such as *Desulfobacteraceae* and *Desulfobulbaceae* (Fig 5). Only a few sequences were classified as *Firmicutes*. Over 80% of the retrieved expressed *pmoA* sequences were affiliated with methanotrophic type Ib *Methylococcaceae* (Fig 5). However, these phyla could not be found by using the 16S rRNA analysis.

31 *nirS* sequences were analyzed, whereby 19% belonged to *Betaproteobacteria*, 42% to *Alphaproteobacteria*, and 29% could only be classified as *Proteobacteria*. Achieved *nirK* sequences were predominantly classified as *Alphaproteobacteria*.

All sequenced active *Archaea* belonged to *Euryarchaeota* classified as *Methanospirillaceae*, *Methanoregulaceae*, *Methanosaetaceae* and *Thermoplasmatales* belonging to the CCA47 group (S7B Fig). Dominant sequences were affiliated with *Methanospirillaceae* (47%) and *Methanoregulaceae* (40%). However, when analyzing the expression of the *mcrA* gene, none was affiliated with the *Methanospirillaceae*, although this family dominated the active *Archaea* community. In contrast, two prominent expressed *mcrA* sequences were distantly related to *Methanoculleus*.

## Discussion

Redox transitions typically occur at aquatic interfaces as the result of the interplay between biogeochemical and physical processes, i.e. stratification due to density gradients or mixing caused by bioturbation or changes in current regimes. These transitions can occur as one-off events or with regularity, reflecting the availability of electron acceptors and donors, with significant effects on specific microbial processes. A well-known example is the SWI of aquatic systems where prominent ORP gradients occur in both space and time [1]. Microbial activities at the SWI affect the exchange of solutes between the overlying water and porewater, and their role in the nutrient balance of aquatic ecosystems is well established [26,27]. In this study we related changes in the hydrochemical conditions at the SWI to changes in the microbial response and vice versa.

### Limitations of DGGE analysis

To avoid methodological biases caused by extracellular DNA in sediments, we focused on the 16S rRNA representing the amount of ribosomes in the cells, and mRNA as an indicator of gene expression. As for all PCR based methods, our results may have been biased by the presence of multiple copies of the target gene, potential primer biases forming multiple or artificial bands on the DGGE gel, or simply a failure of primer binding to potential targets [28–30]. The 16S rRNA analysis via DGGE only gave a partial picture of the active microbial community because organisms of less than 1% of the community may not produce visible DGGE bands [31]. Comparison of 16S rRNA data with representatives of functional gene assays showed only minor similarities, which might be a result of insensitivity of the DGGE system. The lack of gammaproteobacterial and deltaproteobacterial sequences in 16S rRNA analysis was also unexpected since these organisms are common in lake sediments and also in Lake Stechlin ([32,33], Frindte et al., unpublished results). It is possible that the 341f and 907r/803r system did not amplify these sequences, but *in silico* analysis in TestPrime (http://www.arb-silva.de/browser/ssu-122/silva-ref-nr/testprime) did not support this assumption. It is likely that the DGGE fingerprint analysis was too insensitive and/or that diversity of individual taxonomic units was relatively high, so that no visible bands occurred on the DGGE gels. Consequently, 16S rRNA analysis via DGGE might have underestimated the diversity and thus potential changes due to changes in environmental conditions. New high throughput sequencing techniques have the potential to overcome such limitations, but require enormous resources especially if the aim is to analyze the expression of different genes over time.

### Redox transitions and functional gene activity

Denitrification became the dominant process following the depletion of oxygen with a characteristic ORP of -40 mV at three occasions. What differed between those events was the response time of the system to establish a stable Pt-electrode reading. The first ORP drop from 360 to -42 mV took 100 h, whereas after the second DO pulse, the associated ORP decreased from 260 to -40 mV in only 10 h. Apparently, other oxidized electron acceptors like manganese, which is present in Lake Stechlin, buffered the system during the first ORP decline [34]. A similar redox buffer capacity has been suggested previously, e.g., for tropical soils [35,36]. The DGGE banding pattern present under the first oxic phase did not reoccur at the end of the experiment (e.g., bacterial 16S rRNA, *nirS* and *dsrB*) when the system returned to being oxic; thus, it seems that the microbial community did not recover from the intervening anoxic condition. Most likely, the recovery time of the microbial community in the aftermath of a prolonged anoxic phase is the result of high resilience [37] and only a small fraction possesses the ability to react more directly on environmental changes [38]. Since we did not analyze the community composition based on DNA, it is likely that we have overlooked many microorganisms present in a dormant stage in the sediment. 16S rRNA analysis showed that the active bacterial community changed little after 400 hours. Crump et al. [39] identified sulfide as the main driver of the microbial community composition in seasonally anoxic estuarine bottom waters, which also might be true for our simulation experiment. In contrast, expression patterns of some functional genes varied throughout this period (e.g., *dsr*B and *aprA*), indicating a much higher sensitivity towards temporal fluctuations in environmental conditions. However, after re-oxidation, prominent members of the active microbial community under oxic conditions did not reoccur, indicating the disappearance of organisms that are not well adapted or may need more than a few days to recover as has been shown for seasonally anoxic waters [15,39].

Therefore, it is not surprising that induced redox transitions revealed the strongest changes in functional expression patterns of sulfate reducers (*dsrB*, Fig 4) and *Cd1*-dependent nitrite reducers (*nirS*), especially during transition phase 3 when the system shifted from nitrate rich to sulfidic conditions. The high diversity in functional gene expression pattern of sulfate reducers (*dsrB*) during the transition from nitrate to sulfate reduction indicates that this critical redox transition stimulates gene expression and hence activity of sulfate reducers. In contrast, *nirS* was expressed while oxygen was still present indicating the simultaneous occurrence of oxic respiration and denitrification processes [40,41]. On the other hand, addition of nitrate to the system greatly reduced the number of expressed *dsrB* genes suggesting that nitrate addition under anoxic conditions results in reduced *dsrB* gene expression until nitrate depletion. Oxygen did not inhibit *dsrB* gene expression (as observed for *nirS*) indicating that NO_x_ concentrations rather than the redox potential itself was responsible for the observed redox-sensitivity in *dsrB* gene expression [17,41].

### Fluctuation in redox conditions and methane cycling

Concentration of dissolved methane in the overlying water is regulated by the interplay of physical processes (diffusion and ebullition) and methanotrophy. Methanogenesis, the source of biogenic methane, is restricted to the deeper sediment layers that are deplete in sulfate [19,42]. The resulting concentration gradient of dissolved methane between the sediment pore water and overlying water leads to diffusion of methane into the overlying water. In the presence of oxygen, aerobic methanotrophic bacteria efficiently prevent methane enrichment at the SWI, as shown earlier by microprofile measurements in lake sediments [43,44], and is also consistent with our methane measurements during the three oxic phases (Fig 2 at T < 10 h, T = 120–140 h, and T > 620 h). The increase in methane in the overlying water after DO depletion, however, was not uniform, and the resulting accumulation pattern differed from that of sulfide. The presence of nitrate seemed to play a critical role by blocking any release of sulfide at the SWI and by controlling methane release rates. Apparently, the plateau of ~ 0.1 mmol L^-1^ of dissolved methane reached at T = 50–120 h and at T = 190–220 h was the result of anaerobic oxidation of methane (AOM) using nitrate as the electron acceptor [45–47]. The finding that methane increased rapidly at a rate of 50 μmol L^-1^ d^-1^ following the depletion of nitrate at T = 220 h (Fig 2) strongly indicates the presence of AOM. In line with this notion, the expressed *pmoA* sequences mainly belonged to *Methylococcaceae* and, surprisingly, they did not vary much during the transition from oxic to anoxic conditions as long as nitrate was present. In our experiment, the presence of oxygen might be an explanation for effective aerobic methane oxidation at the beginning of the experiment, but this is unlikely when ORP decreased to -40 mV. In this case, AOM using oxidized nitrogen species as electron acceptors offers the most likely explanation [45,46]. From the parallel increase of sulfide and methane at T > 220 h (Fig 2), we may conclude that methanotrophs in our experiment were incapable of AOM with sulfate, a process common in marine sediments [48]. Apparently, the sediments in the oligotrophic Lake Stechlin harbour the potential for AOM via NO_x_ compounds despite the very low ambient nitrate and nitrite concentrations [46,47]. The addition of nitrate at T = 400 h caused a fast depletion of sulfide. We calculated an oxidation rate of sulfide with nitrate of 50 μmol L^-1^ h^-1^, which is equivalent to that with oxygen. Methane concentration, on the other hand, showed a gradual decline with nitrate as the electron acceptor at a rate of 1.3 μmol L^-1^ h^-1^ until nitrate depletion. Taking into consideration the measured methane release rate of 2.5 μmol L^-1^ h^-1^, methane oxidation rates with nitrate were 3.8 μmol L^-1^ h^-1^ while under oxic conditions they reached ca. 12.5 μmol L^-1^ h^-1^ –still five times lower than for sulfide. Thus, it seems likely that sulfide oxidation with nitrate is energetically more efficient than AOM with nitrate. This is consistent with the findings by Ettwig et al. [46], who posited that AOM with NO_x_ compounds is mainly nitrite dependent, and needs the denitrification steps to procure nitrite for the intracellular oxygen production.

## Conclusions

The goal of the present study was to link redox transitions in the overlying water of an intact sediment core to changes in microbial activity. Using a combination of continuous monitoring of the critical chemical parameters and the frequent analysis of specific functional genes, our approach enabled us to follow closely the microbial response to an altering electron acceptor availability. Our results show that during several transitions from oxic-heterotrophic to sulfate-reducing conditions, especially nitrate-availability and the on-set of sulfate reduction, critically affected the corresponding functional gene expression in free-living and particle associated microorganisms at the SWI. Evidence for anaerobic methane oxidation with NO_x_ is presented. Overall our research provides new insight into the temporal dynamics of the interplay between microbial activity and redox processes at oxic–anoxic interfaces as they typically occur in stratified zones of aquatic systems.

## Supporting Information

S1 Fig Location and classification of DGGE bands using 16S rRNA bacterial primer pair 341f-gc and 803r. Numbers above bars refer to time points of sampling.
(TIF)

S2 Fig DGGE picture of PCR products amplified with 16S archaea primers. Temporal changes in dominat bands were minor. Numbers above bars refer to time points of sampling.
(TIF)

S3 Fig DGGEs bands using specific primers. (**A)** dsrB and (**B)** nirS. Numbers on bottom of bars plates refer to time points of sampling.
(TIF)

S4 Fig Cluster analysis of *nirS* expression patterns in a nitrate adaption experiment. A core was treated with barium chloride to lower sulfate reduction activity. This experiment was performed to examine the immediate effects of nitrate addition on *nirS* gene expression only minutes after nitrate addition. 40 mg L^-1^ nitrate was added and samples for molecular analysis were taken prior nitrate addition and after 5, 15, 30, 45 and 60 minutes.
(TIF)

S5 Fig Overview on changes in DGGE band diversity of different genes evaluated in this study. (TIF)

S6 Fig Development of chemical parameters in a pilot study in 2007. Methane and S[-II] values increased only after oxygen and nitrate depletion.
(TIF)

S7 Fig Community structure of microorganisms at the sediment-water-interface. Phylogenetic classification of 32 bacterial and 15 archaeal 16S rRNA sequences on phylum/class and family level. (A) Affiliation of 16S rRNA sequences to phyla and respective families. (B) Affiliation of 16S rRNA archaeal sequences.
(TIF)

S1 Table Used primer systems and DGGE conditions. Ref. = Reference, AA = Acrylamide
(DOCX)

S2 Table Summary of the statistic results from envfit algorithm within the NMDS plots. The *p* values are given, all significant values are italicized. All values still significant after Bonferroni-Holm correction are written in bold and italic letters. Furthermore, stress values of NMDS plots are given.
(DOCX)
