# Supplementary figures and images for: Microbial Response to Experimentally Controlled Redox Transitions at the Sediment Water Interface
Source: PLoS One. 2015 Nov 24;10(11):e0143428. doi: 10.1371/journal.pone.0143428 (PMC4657962; doi:10.1371/journal.pone.0143428)

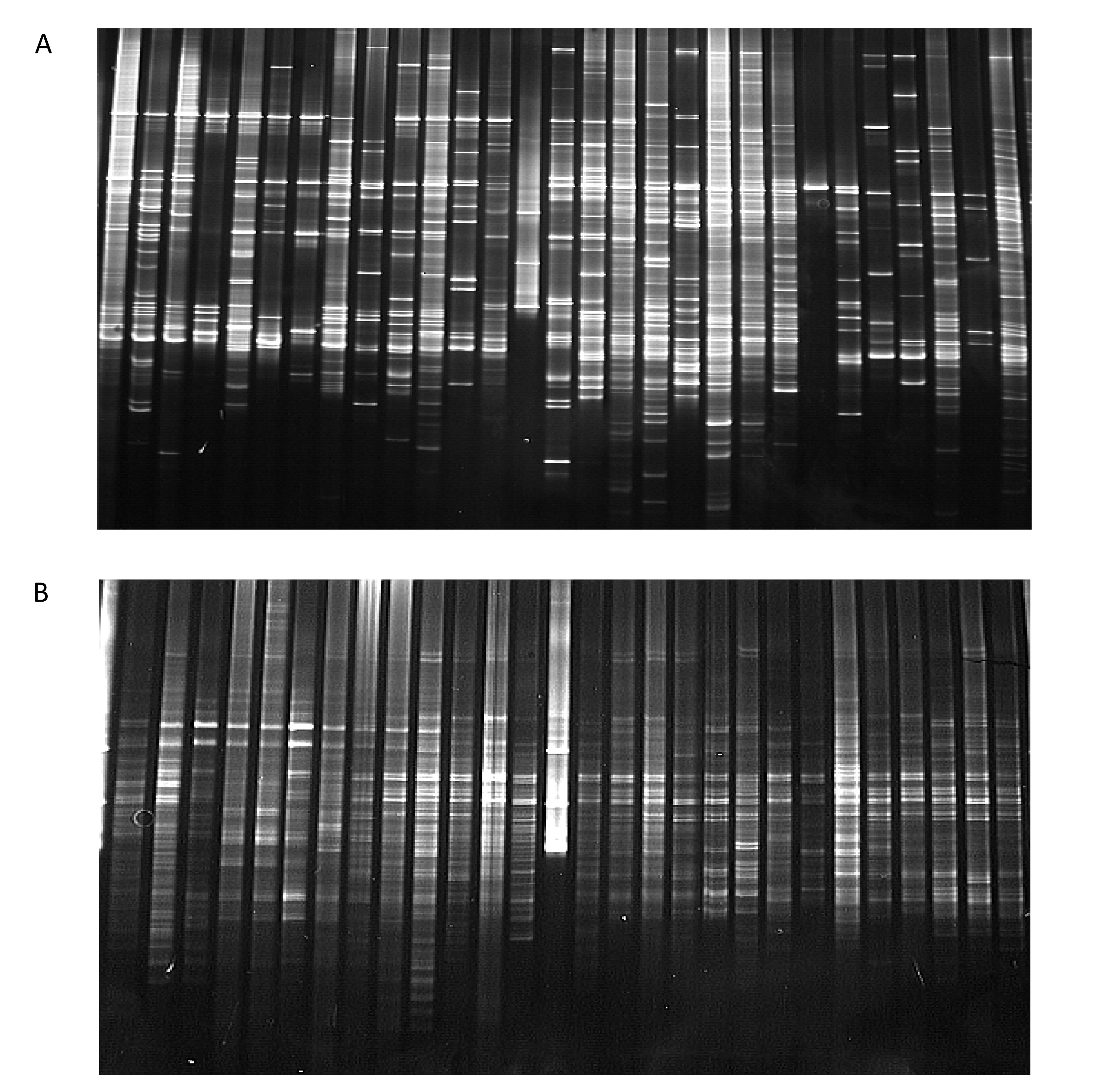

Supplement: S3 Fig — (A) dsrB and (B) nirS. Numbers on bottom of bars plates refer to time points of sampling. (TIF) [file pone.0143428.s003.tif]

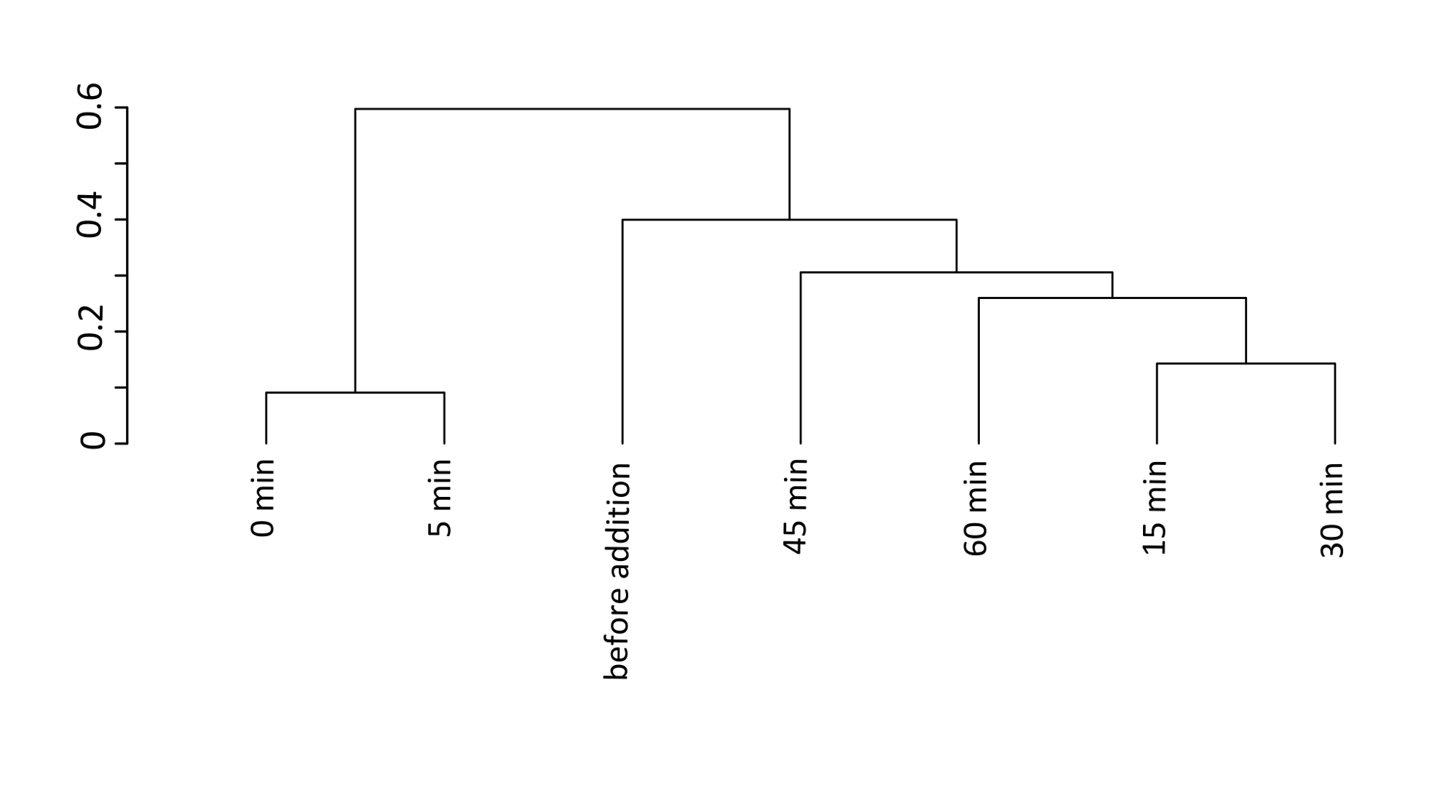

Supplement: S4 Fig — A core was treated with barium chloride to lower sulfate reduction activity. This experiment was performed to examine the immediate effects of nitrate addition on nirS gene expression only minutes after nitrate addition. 40 mg L-1 nitrate was added and samples for molecular analysis were taken prior nitrate addition and after 5, 15, 30, 45 and 60 minutes. (TIF) [file pone.0143428.s004.tif]

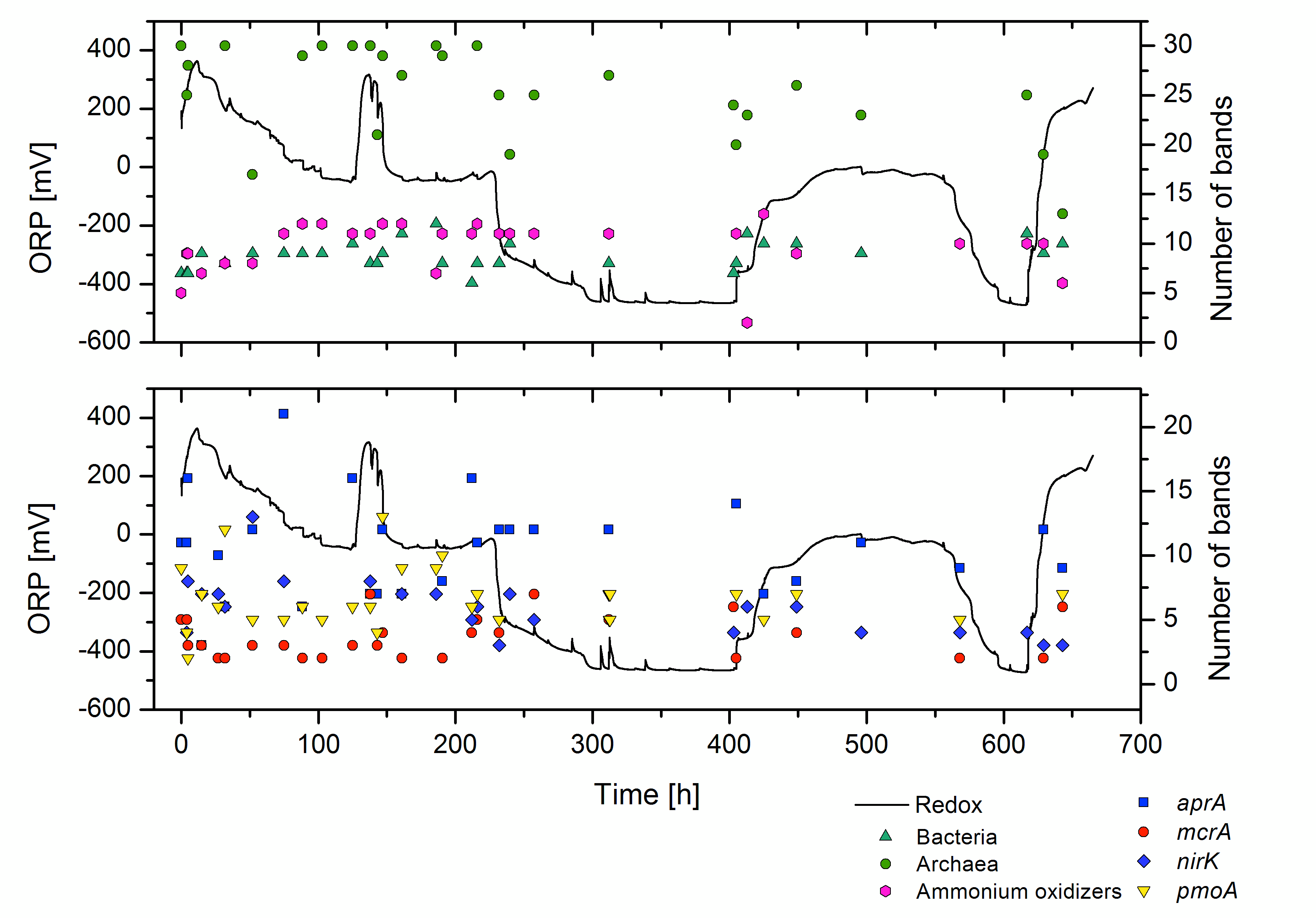

Supplement: S5 Fig — (TIF) [file pone.0143428.s005.tif]

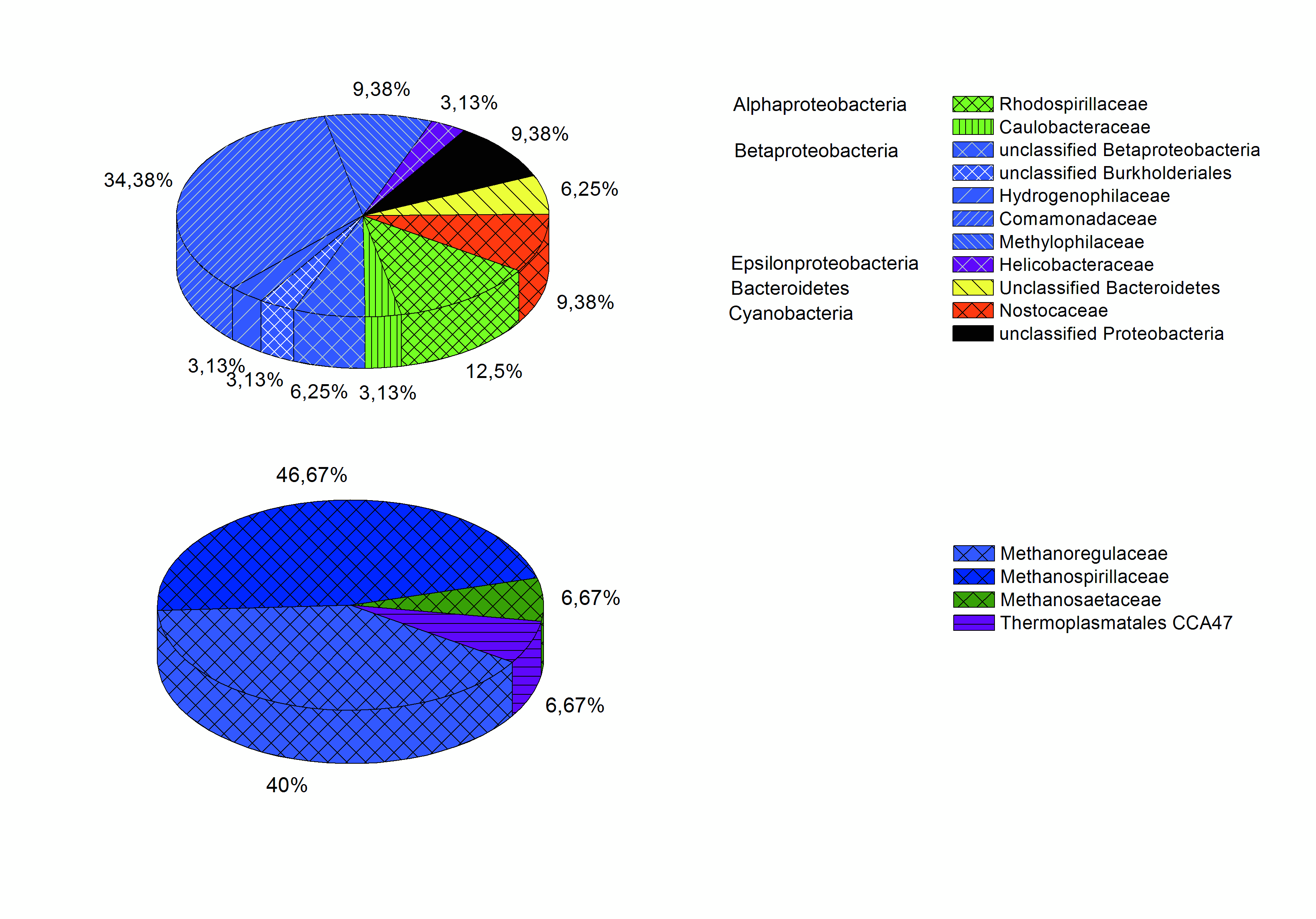

Supplement: S7 Fig — Phylogenetic classification of 32 bacterial and 15 archaeal 16S rRNA sequences on phylum/class and family level. (A) Affiliation of 16S rRNA sequences to phyla and respective families. (B) Affiliation of 16S rRNA archaeal sequences. (TIF) [file pone.0143428.s007.tif]
