# Supplementary material for: Microbial Response to Experimentally Controlled Redox Transitions at the Sediment Water Interface
Source: PLoS One. 2015 Nov 24;10(11):e0143428. doi: 10.1371/journal.pone.0143428 (PMC4657962; doi:10.1371/journal.pone.0143428)
Supplement: S1 Table — Ref. = Reference, AA = Acrylamide (DOCX) [file pone.0143428.s008.docx]

**Table S1:** Used primer systems and DGGE conditions (Ref. = Reference, AA= Acrylamide)

| **Primer** | **Function** | **Sequence** | **Program** | **Ref.** | **Gradient**  **AA** |
| --- | --- | --- | --- | --- | --- |
| **341f***  **907r**  **803** | 16S *Bacteria* | CCT ACG GGA GGC AGC AG  CCG TCA ATT CMT TTG AGT TT  CTA CCA GGG TAT CTA ATC C | 95°C 10 min; 1 min 95°C, 1 min 55°C, 1 min 72°C (35x); 10 min 72°C | [1–3] | 40-65  7% |
| **357f***  **691r** | 16S *Archaea* | GGA TTA CAR GAT TTC AC  CCC TAC GGG GCG CAG CAG | 95°C 5 min; 45s 95°C, 45s 54°C, 45s 72°C (35x); 10 min 72°C | [4] | 40-65  7% |
| **189f***  **654r** | 16S ammonium oxidizer | GGA GRA AAGYAG GGG ATC G  CTA GCY TTG TAGTTT CAA ACG C | 95°C 5 min; 45s 95°C, 45s 58°C, 45s 72°C (40x); 10 min 72°C | [5] | 40-65  7% |
| ***dsr*4r**  **P2060f***  (300 bp) | Dissimilatory sulfite reductase (*dsrB*) | GTG TAG CAG TTA CCG CA  CAA CAT CGT YCA YAC CCA GGG | 95°C 5 min, 45 s 95°C, 45s 56°C, 45 s 72°C (35x), 10 min 72°C | [6, 7] | 40-70  7% |
| ***aprA* 1f***  ***aprA* 5r**  (400 bp) | Dissimilatory adenosine-5-phosphosulfate reductase (*aprA*) | TGG CAG ATC ATG ATY MAY GG  GCG CCA ACY GGR CCR TA | 95°C 5 min; 45 s 95°C, 45 s 55°C, 45 s 72°C (40x); 10 min 72°C | [8] | 40-65  8% |
| **Me1f#**  **Me2r**  **Me3f***  (500 bp) | Methyl-coenzyme M reductase (*mcrA*) | GCM ATG CAR ATH GGW ATG TC  TCA TBG CRT AGT TDG GRT AGT  ATG TCN GGT GGH GTM GGS TTY AC | 95°C 5 min; 45 s 95°C, 45 s 50°C, 1.5 min 72°C (35 cycles); 10 min 72°C  # 95°C 5 min; 45s 95°C, 45s 54°C, 45s 72°C (40x); 10 min 72°C | [9, 10] | 40-70  8% |
| ***nirS* cd3af**  ***nirS R*3cd****  (500 bp) | Cytochrome *cd*_1_-depending nitrite reductase (*nirS*) | GTS AAC GTS AAG GAR ACS GG  GAS TTC GGR TGS GTC TTG A | 95°C 10 min; 45 s 95°C, 45s 60°C, 45 s 72°C (40x); 10 min 72°C | [11] | 40-70  8% |
| ***nirK* FlaCu**  ***nirKR*3Cu****  (450 bp) | Copper-depending nitrite reductase (*nirK*) | ATC ATG GTS CTG CCG CG  GCC TCG ATC AGR TTG TGG TT | 95°C 10 min; 45 s 95°C, 45s 65°C, 45 s 72°C (40x); 10 min 72°C | [11] | 40-70  8% |
| **A189f#**  **682r**  **mb661_nd** | Particulate  methane monooxygenase (*pmoA*) | GGN GAC TGG GAC TTC TGG  GAA SGC NGA GAA GAA SGC  CCG GCG CAA CGT CCT TAC C | 95°C 10 min; 45 s 95°C, 45s 61°C, 1min 72°C (35x); 10 min 72°C  # 95°C 10 min; 45 s 95°C, 45s 57°C, 1 min 72°C (35x); 10 min 72°C | [12, 13] | 35-65  7% |

** GC clamp: CGC CCG CCG CGC CCC GCG CCC GTC CCG CCG CCC CCG CCC G*

***GC clamp: GGC GGC GCG CCG CCC GCC CCG CCC CCG TCG CCC G*

*# semi-nested approach, first with Me1f and Me2r, second with Me2r and Me3f-gc, pmoA: first 189f und 682r, second 189f and mb661_nd*
