# Supplementary material for: Microbial Response to Experimentally Controlled Redox Transitions at the Sediment Water Interface
Source: PLoS One. 2015 Nov 24;10(11):e0143428. doi: 10.1371/journal.pone.0143428 (PMC4657962; doi:10.1371/journal.pone.0143428)
Supplement: S2 Table — The p values are given, all significant values are italicized. All values still significant after Bonferroni-Holm correction are written in bold and italic letters. Furthermore, stress values of NMDS plots are given. (DOCX) [file pone.0143428.s009.docx]

|  | **Dissolved oxygen** | **ORP** | **Nitrate** | **Sulfide** | **Methane** | **pH** | **NMDS stress value** |
| --- | --- | --- | --- | --- | --- | --- | --- |
| **16S rRNA,** *Bacteria* | 0.128 | 0.159 | ***0.007*** | 0.281 | ***0.002*** | 0.061 | 0.13 |
| **16S rRNA,** *Archaea* | ***0.008*** | ***0.001*** | ***0.001*** | 0.267 | ***0.006*** | 0.095 | 0.16 |
| **16S rRNA,** NH_4_^+^-oxidizers | 0.078 | ***0.009*** | ***0.001*** | 0.706 | *0.028* | *0.037* | 0.07 |
| ***nirS*,** denitrification | 0.101 | ***0.006*** | ***0.016*** | 0.174 | ***0.001*** | 0.542 | 0.20 |
| ***nirK*,** denitrification | 0.422 | 0.130 | 0.100 | 0.469 | 0.749 | 0.771 | 0.15 |
| ***dsrB*,** sulfate reduction | 0.463 | ***0.007*** | *0.048* | 0.950 | ***0.010*** | 0.431 | 0.19 |
| ***aprA*,** sulfate reduction /sulfide oxidation | 0.089 | ***0.011*** | *0.026* | 0.208 | ***0.001*** | 0.327 | 0.20 |
| ***mcrA*,** methanogenesis | *0.040* | 0.819 | 0.691 | 0.390 | 0.277 | 0.749 | 0.13 |
| ***pmoA*,** methanotrophic bacteria | 0.168 | 0.215 | *0.028* | 0.521 | 0.337 | ***0.006*** | 0.17 |

**Table S2:** Table 2: Summary of the statistic results by using envfit algorithm within NMDS plot. The *p* values are given, all significant values are italicized. All values still significant after Bonferroni-Holm correction are written in bold and italic letters. Furthermore, stress values of NMDS plots are given.
